# Home-based modified constraint-induced movement therapy improves distal motor outcomes after botulinum toxin A injection in chronic stroke

**DOI:** 10.3389/fneur.2026.1906566

**Published:** 2026-09-07

**Authors:** Wenjing Zhang, Shuya Mei, Min Wen, Kai Jiao, Langfang Zhao, Rui Zhang

**Affiliations:** Department of Neurological Rehabilitation I, Xi’an International Medical Center Hospital, Xi’an, Shaanxi, China

**Keywords:** Botulinum toxin type A, chronic stroke, finger flexor spasticity, home-based modified constraint-induced movement therapy, upper-limb rehabilitation

## Abstract

**Background:**

Botulinum toxin type A (BoNT-A) effectively reduces focal spasticity. However, this pharmacological effect often fails to translate into consistent functional gains when patients receive conventional rehabilitation after injection.

**Methods:**

Clinical data from 102 patients with chronic stroke and finger flexor spasticity who received ultrasound-guided BoNT-A injections were retrospectively analyzed in a single-center cohort study. Patients were categorized into home-based modified constraint-induced movement therapy (h-mCIMT) or dose-matched conventional home-based occupational therapy (h-COT) according to post-injection rehabilitation strategies documented in clinical records. In routine clinical practice, rehabilitation was initiated around day 3 after injection and performed 5 days per week for 4 weeks. Clinical outcomes, including distal FMA-UE, ARAT, MAL, and MAS, were extracted from routine assessments at baseline and follow-up visits.

**Results:**

Of the 102 eligible patients, 98 had complete follow-up outcome records and were included in final analysis. Both cohorts exhibited comparable reductions in spasticity, suggesting similar pharmacological effects of BoNT-A. Compared with the h-COT cohort, the h-mCIMT cohort achieved significantly greater distal motor recovery at 4 weeks and 3 months, indicating superior functional hand performance. This was further reflected in greater real-world use of the affected limb, with significantly higher MAL scores at both follow-up assessments.

**Conclusion:**

Home-based modified constraint-induced movement therapy following BoNT-A injection provides a feasible and effective strategy to enhance distal motor recovery and real-world upper-limb use in chronic stroke. This combined intervention underscores the importance of structured, task-oriented rehabilitation during the post-injection therapeutic window as a key mechanism for maximizing functional recovery.

## 1 Introduction

Stroke remains one of the leading causes of long-term disability worldwide, affecting more than 100 million individuals globally, with a continuously increasing incidence in aging populations (1, 2). Upper-limb motor impairment, particularly hand dysfunction caused by finger flexor spasticity, is highly prevalent and significantly limits activities of daily living and quality of life (3). Botulinum toxin type A (BoNT-A) injection has been widely adopted as an effective pharmacological intervention for focal spasticity management and is recommended in multiple clinical guidelines (4–6). However, despite its widespread use, the translation of spasticity reduction into meaningful functional recovery remains suboptimal, highlighting an urgent clinical need for optimized post-injection rehabilitation strategies (7, 8).

Current rehabilitation approaches after BoNT-A injection remain limited by several challenges. Conventional therapy often focuses on passive range-of-motion exercises and general occupational training, which may not sufficiently promote task-specific motor relearning or overcome learned non-use of the affected limb (9). In addition, variability in rehabilitation intensity, poor treatment adherence in home settings, and lack of standardized post-injection protocols further contribute to inconsistent functional outcomes. As a result, a persistent gap exists between impairment-level improvement and real-world functional use of the upper limb, particularly in chronic stroke populations.

Home-based modified constraint-induced movement therapy (h-mCIMT) has emerged as a promising approach to address these limitations by combining task-oriented training with constraint of the unaffected limb, thereby reinforcing use-dependent cortical reorganization (10–13). Importantly, the early post-BoNT-A period may represent a critical therapeutic window during which reduced spasticity can facilitate motor relearning and functional engagement (14). However, whether structured home-based h-mCIMT during this window can enhance the functional translation of BoNT-A remains insufficiently explored in real-world clinical settings (15).

Therefore, this study retrospectively analyzed clinical data from chronic stroke patients receiving BoNT-A injection followed by either h-mCIMT or conventional home-based occupational therapy. We aimed to compare motor recovery, upper-limb functional performance, and real-world arm use between the two rehabilitation strategies. By integrating pharmacological spasticity reduction with structured behavioral training, this study sought to provide real-world evidence for an optimized, feasible, and scalable post-BoNT-A rehabilitation strategy.

## 2 Method

### 2.1 Study design

This retrospective cohort study analyzed clinical data from patients with chronic stroke and finger flexor spasticity. All patients received BoNT-A injections at a tertiary rehabilitation center between January 2025 and December 2025. According to routine clinical rehabilitation strategies following BoNT-A treatment, patients were categorized into two groups: those who received h-mCIMT and those who received conventional home-based occupational therapy (h-COT). The study was approved by the Institutional Review Board of Xi’an International Medical Center Hospital (No. GJYX-KY-2024-047).

### 2.2 Data source and participants

Medical records were extracted from the hospital electronic medical record system and rehabilitation follow-up database. Patients were then evaluated according to predefined eligibility criteria. Inclusion criteria were: (1) first-ever stroke confirmed by CT or MRI; (2) disease duration ≥6 months; (3) age 18–80 years; (4) finger flexor spasticity with Modified Ashworth Scale (MAS) ≥ 2 (16); (5) Brunnstrom stage III or higher for the affected upper limb; (6) ability to sit independently for at least 30 min; (7) Cognitive function was screened using the Mini-Cog (a 0–5 point scale; score ≥3 indicating normal cognition) (17); and (8) availability of caregiver-supported home rehabilitation records. Exclusion criteria included prior BoNT-A injection or nerve block within 6 months, fixed contracture or severe deformity of the affected limb, severe aphasia or neglect affecting rehabilitation participation, major comorbid neurological or systemic diseases, or incomplete clinical data.

### 2.3 Post-injection rehabilitation strategies

Patients were classified into two cohorts based on rehabilitation strategies documented in routine medical and rehabilitation records following BoNT-A injection. Individuals who received h-mCIMT were identified as the h-mCIMT cohort, whereas those who received conventional h-COT were identified as the h-COT cohort. Rehabilitation strategies were implemented as part of routine clinical care and were not assigned for research purposes. In the h-mCIMT cohort, the less-affected upper limb was restrained using a mitt for approximately 4 h per day during waking hours, 5 days per week, for 4 weeks. The restraint period extended beyond the 60-min structured task-oriented training session. During each session, patients performed repetitive grasping, releasing, pinching, object transfer, dressing, feeding-related activities, and other functional hand tasks. Training tasks included repetitive grasping, releasing, pinching, object transfer, dressing, feeding-related activities, and other functional hand tasks. Caregiver-assisted home practice was monitored using daily training logs and weekly video-based follow-ups to ensure adherence and provide corrective feedback. In the h-COT cohort, patients received conventional home-based occupational therapy consisting of passive range-of-motion exercises, stretching, bilateral reaching activities, gross upper-limb movement training, and general activities of daily living without limb restraint. Training frequency and duration were generally matched to routine rehabilitation recommendations. Both cohorts received ultrasound-guided BoNT-A injections using standardized clinical protocols, and rehabilitation intensity and duration were recorded as part of routine post-injection management.

### 2.4 BoNT-A injection protocol

All patients received ultrasound-guided BoNT-A injections targeting the flexor digitorum superficialis, flexor digitorum profundus, and flexor pollicis longus. These muscles were selected to reduce excessive finger flexion and facilitate hand opening and grasp release; the wrist flexors were not injected, and separate standardized measurements of wrist flexor spasticity were not systematically available. Injection doses were individualized according to spasticity severity and body weight, with a total dose not exceeding 300 U. Procedures were performed by experienced rehabilitation physicians with at least 8 years of clinical expertise. Post-injection recommendations, including avoidance of strenuous activity for 24 h, were uniformly provided.

### 2.5 Outcome measures

Outcome data were extracted from routine clinical assessments recorded at three time points: baseline (T0), approximately 4 weeks after BoNT-A injection (T1), and 3 months after injection (T2). The Primary outcome was the distal subscore of the Fugl-Meyer Assessment of the Upper Extremity (FMA-UE), which evaluates wrist and hand motor function (0–30 points). Higher scores indicate better motor recovery. Baseline total FMA-UE score was used to characterize the overall severity of upper-limb motor impairment, whereas the distal FMA-UE subscore was selected as the primary outcome because the intervention principally targeted wrist and hand function. Baseline distal FMA-UE scores were also compared separately to assess between-group comparability in the motor domain corresponding to the primary outcome. Secondary outcomes included: (1) Total and proximal FMA-UE scores; (2) Modified Ashworth Scale (MAS) for finger flexor spasticity; (3) Action Research Arm Test (ARAT) for upper-limb functional ability; (4) Motor Activity Log (MAL), including amount of use (AOU) and quality of movement (QOM). Adverse events documented in medical records were extracted, including injection-related reactions, training-related fatigue or pain, and other reported clinical events.

### 2.6 Statistical analysis

All statistical analyses were performed using SPSS version 26.0 and SAS version 9.4. Continuous variables were expressed as mean ± standard deviation or median (interquartile range), depending on distribution. Categorical variables were presented as frequencies and percentages. Baseline differences between groups were assessed using independent-samples *t*-tests, Mann–Whitney *U* tests, or chi-square tests as appropriate. To account for potential selection bias inherent in retrospective cohort design, multivariable linear regression models were used to adjust for baseline covariates including age, sex, stroke duration, baseline FMA-UE score, Brunnstrom stage, and MAS grade. In sensitivity analyses, generalized estimating equations (GEE) were applied to examine time, group, and time-by-group interaction effects across repeated measurements. Patients with incomplete follow-up outcome data were excluded from the final analytic cohort. Because only four patients had incomplete outcome records (3.9%), complete-case analysis was considered appropriate. Statistical significance was defined as a two-sided *p* value <0.05. No formal *a priori* sample-size calculation was performed because all consecutive patients who met the eligibility criteria during the predefined study period were included. Therefore, the sample size was determined by the number of eligible cases available rather than by a prespecified effect size or an exploratory pilot-trial feasibility target.

## 3 Results

### 3.1 Subject recruitment process and baseline characteristics

Between January 2025 and December 2025, a total of 186 patients with post-stroke finger flexor spasticity were identified from the hospital electronic medical records. After applying the predefined eligibility criteria, 65 patients were excluded, mainly due to stroke duration <6 months, Brunnstrom stage <III, or Mini-Cog score <3. An additional 19 patients were excluded because of incomplete medical or rehabilitation records. Thus, 102 patients were initially included in the cohort. Based on rehabilitation strategies documented in routine clinical records following BoNT-A injection, patients were classified into the h-mCIMT cohort (*n* = 51) and the h-COT cohort (*n* = 51). During subsequent outcome record review, two patients in the h-mCIMT cohort and two in the h-COT cohort were excluded due to incomplete follow-up outcome data. The final analytic cohort consisted of 98 patients, with 49 patients in each cohort. Baseline demographic and clinical characteristics of the two cohorts are summarized in Table 1. There were no statistically significant differences between cohorts in age, sex, disease duration, stroke type, affected side, Brunnstrom stage, baseline MAS grade, total BoNT-A dose, or baseline upper-limb functional scores, including total FMA-UE, proximal FMA-UE, distal FMA-UE, ARAT, MAL-AOU, and MAL-QOM (all *p* > 0.05). These findings indicate that the two cohorts were comparable at baseline.

**Table 1:** Comparison of baseline demographic and clinical characteristics between the h-mCIMT and h-COT cohorts.

Indicator	h-mCIMT cohort (*n* = 49)	h-COT cohort (*n* = 49)	*t*/*χ*^2^/*Z* value	*p*value
Age (years)	59.82 ± 10.46	60.14 ± 11.02	−0.147	0.883
Sex (male/female, *n*)	31/18	29/20	0.174	0.677
Disease duration (months)	16.24 ± 10.38	15.86 ± 9.94	0.186	0.853
Stroke type (ischemic/hemorrhagic, *n*)	36/13	38/11	0.233	0.630
Hemiplegic side (left/right, *n*)	23/26	24/25	0.04	0.842
Brunnstrom stage (upper limb, III/IV/V/VI)	12/22/12/3	13/21/13/2	0.311	0.958
Finger flexor MAS score [median (P25, P75)]	3.0 (2.0, 3.0)	3.0 (2.0, 3.0)	−0.426	0.670
Total FMA-UE score	30.34 ± 7.82	29.95 ± 7.64	0.248	0.805
FMA-UE proximal	17.08 ± 4.62	16.94 ± 4.48	0.153	0.879
FMA-UE distal score	13.26 ± 3.41	13.01 ± 3.38	0.365	0.716
Total ARAT score	22.46 ± 8.16	22.14 ± 7.94	0.198	0.844
MAL-AOU	0.58 ± 0.31	0.55 ± 0.29	0.497	0.620
MAL-QOM	0.51 ± 0.28	0.49 ± 0.27	0.363	0.717
Total BoNT-A dose (U)	176.32 ± 42.18	173.86 ± 40.65	0.295	0.769

### 3.2 Spasticity assessed by MAS

At 4 weeks after BoNT-A injection (T1), MAS scores for the finger flexors were significantly reduced from baseline (T0) in both cohorts (Table 2). At the 3-month follow-up (T2), MAS scores had partially increased compared with T1 but remained significantly lower than baseline. No significant between-cohort differences in MAS scores were observed at any time point. GEE analysis showed a significant main effect of time (Wald *χ*^2^ = 142.356, *p* < 0.001), but no significant cohort effect (Wald *χ*^2^ = 0.218, *p* = 0.641) or cohort-by-time interaction (Wald *χ*^2^ = 0.864, *p* = 0.649). These findings suggest that both rehabilitation strategies were associated with comparable reductions in finger flexor spasticity following BoNT-A injection.

**Table 2:** Changes in MAS scores over time.

Outcome/time point	h-mCIMT cohort (*n* = 49)	h-COT cohort (*n* = 49)	Between-group *p* value
T0	3.0 (2.0, 3.0)	3.0 (2.0, 3.0)	0.670
T1	1.0 (1.0, 2.0)	1.0 (0.5, 2.0)	0.586
T2	1.5 (1.0, 2.0)	1.5 (1.0, 2.0)	0.482

### 3.3 Changes in FMA-UE scores

Both cohorts showed improvement in FMA-UE total scores after BoNT-A injection and subsequent rehabilitation; however, the magnitude of improvement was greater in the h-mCIMT cohort (Table 3). At T1, the between-cohort difference in FMA-UE total score was 4.22 points (95% CI, 0.96–7.48; *p* = 0.012) in favor of the h-mCIMT cohort, and this difference remained significant at the 3-month follow-up (*p* = 0.008). In contrast, proximal FMA-UE subscores improved modestly in both cohorts, with no significant between-cohort difference at either the 4-week or 3-month assessment or follow-up assessment.

**Table 3:** Changes in FMA-UE scores over time.

FMA-UE score	h-mCIMT cohort (*n* = 49)	h-COT cohort (*n* = 49)	Between-group *p* value
Total FMA-UE score (score)			
T0	30.34 ± 7.82	29.95 ± 7.64	0.805
T1	38.42 ± 8.36	34.20 ± 7.88	0.012
T2	37.26 ± 8.22	32.86 ± 7.92	0.008
Proximal FMA-UE score (score)			
T0	17.08 ± 4.62	16.94 ± 4.48	0.879
T1	18.60 ± 4.74	18.26 ± 4.52	0.694
T2	18.54 ± 4.68	17.08 ± 4.42	0.116
Distal FMA-UE score (score)			
T0	13.26 ± 3.41	13.01 ± 3.38	0.716
T1	19.82 ± 4.52	15.94 ± 4.28	<0.001
T2	18.72 ± 4.46	14.78 ± 4.16	<0.001

**Figure 1:**
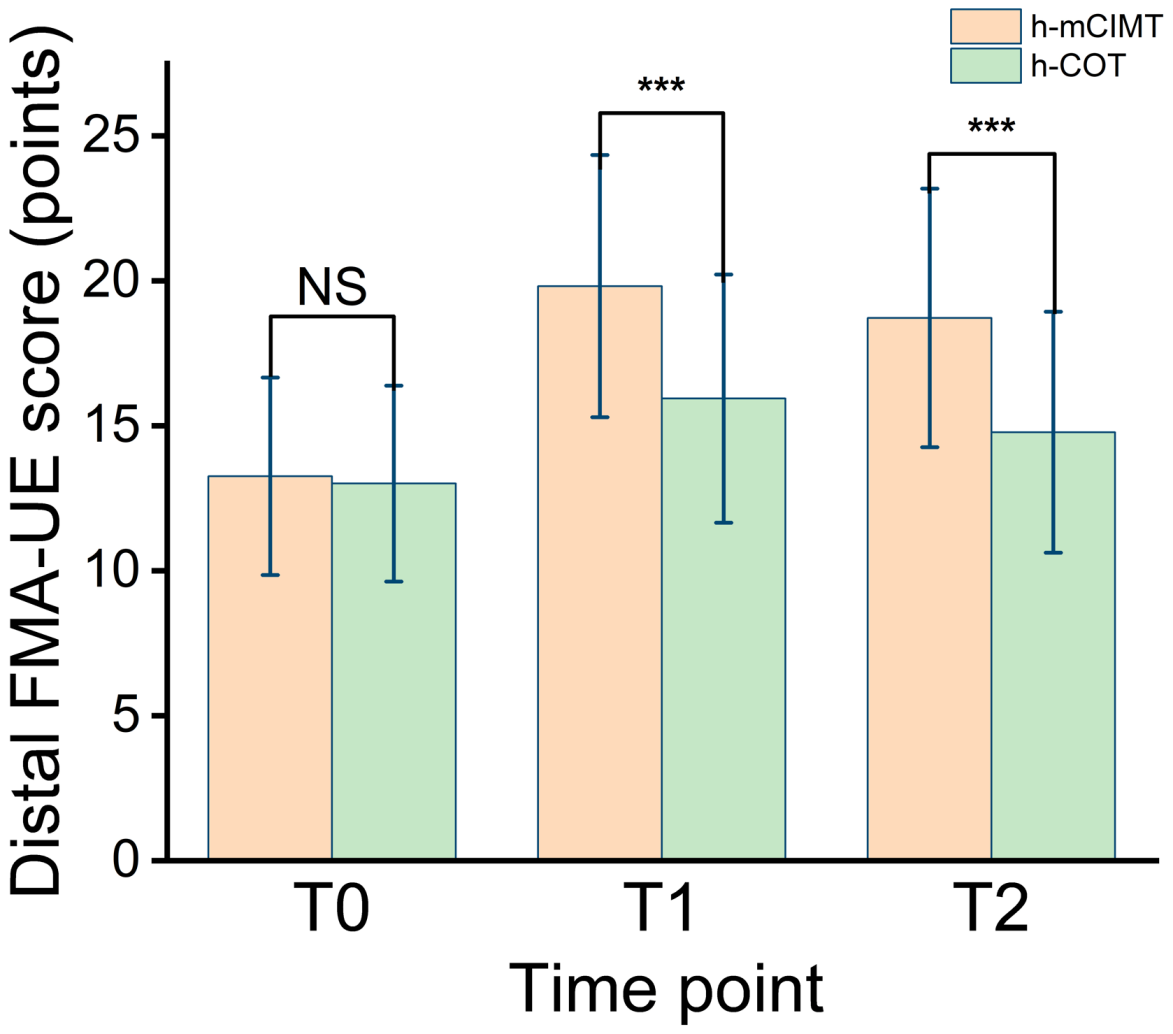
Changes in distal FMA-UE scores over time.

The trajectories of distal FMA-UE are shown in Figure 1. The most prominent functional improvement was observed in the distal FMA-UE subscore, which served as the primary outcome of this study. At T1, the h-mCIMT cohort showed a substantially greater gain in distal motor function than the h-COT cohort, with a between-cohort difference of 3.88 points (95% CI, 2.11–5.67; *p* < 0.001). After adjustment for age, sex, stroke duration, baseline FMA-UE score, Brunnstrom stage, and MAS grade, this between-cohort difference remained significant (adjusted mean difference, 3.71 points; 95% CI, 2.03–5.39; *p* < 0.001). This advantage was maintained at the 3-month follow-up, with a between-cohort difference of 3.94 points (95% CI, 2.21–5.67; *p* < 0.001), indicating persistence of the observed functional difference beyond the initial rehabilitation phase.

GEE analysis further confirmed a significant cohort-by-time interaction for the distal FMA-UE subscore (Wald *χ*^2^ = 18.246, *p* < 0.001). Sensitivity analyses yielded findings consistent with the primary analysis. Taken together, these findings suggest that h-mCIMT following BoNT-A injection was associated with a selective and sustained improvement in distal upper-limb motor recovery, rather than a generalized improvement across all upper-limb segments.

### 3.4 Changes in ARAT scores

ARAT scores improved from baseline to T1 in both cohorts, indicating that BoNT-A combined with home-based rehabilitation was associated with improved upper-limb activity capacity (Table 4). Although the h-mCIMT cohort showed a numerically greater improvement than the h-COT cohort, the between-cohort difference at T1 did not reach statistical significance (mean difference, 2.92 points; 95% CI, −0.64 to 6.48; *p* = 0.107). At T2, ARAT scores were largely maintained in the h-mCIMT cohort but declined modestly in the h-COT cohort; however, the between-cohort difference remained non-significant (*p* = 0.061). These findings suggest a favorable trend toward better functional arm activity in the h-mCIMT cohort, although the present cohort size may have limited the statistical power to detect significant between-cohort differences in ARAT outcomes.

**Table 4:** Comparison of ARAT scores between the two cohorts at three time points.

ARAT score (score)	h-mCIMT cohort (*n* = 49)	h-COT cohort (*n* = 49)	Between-cohort *p* value
T0	22.46 ± 8.16	22.14 ± 7.94	0.844
T1	30.24 ± 9.02	27.32 ± 8.72	0.107
T2	28.82 ± 8.94	25.44 ± 8.64	0.061

### 3.5 Changes in MAL scores

**Figure 2:**
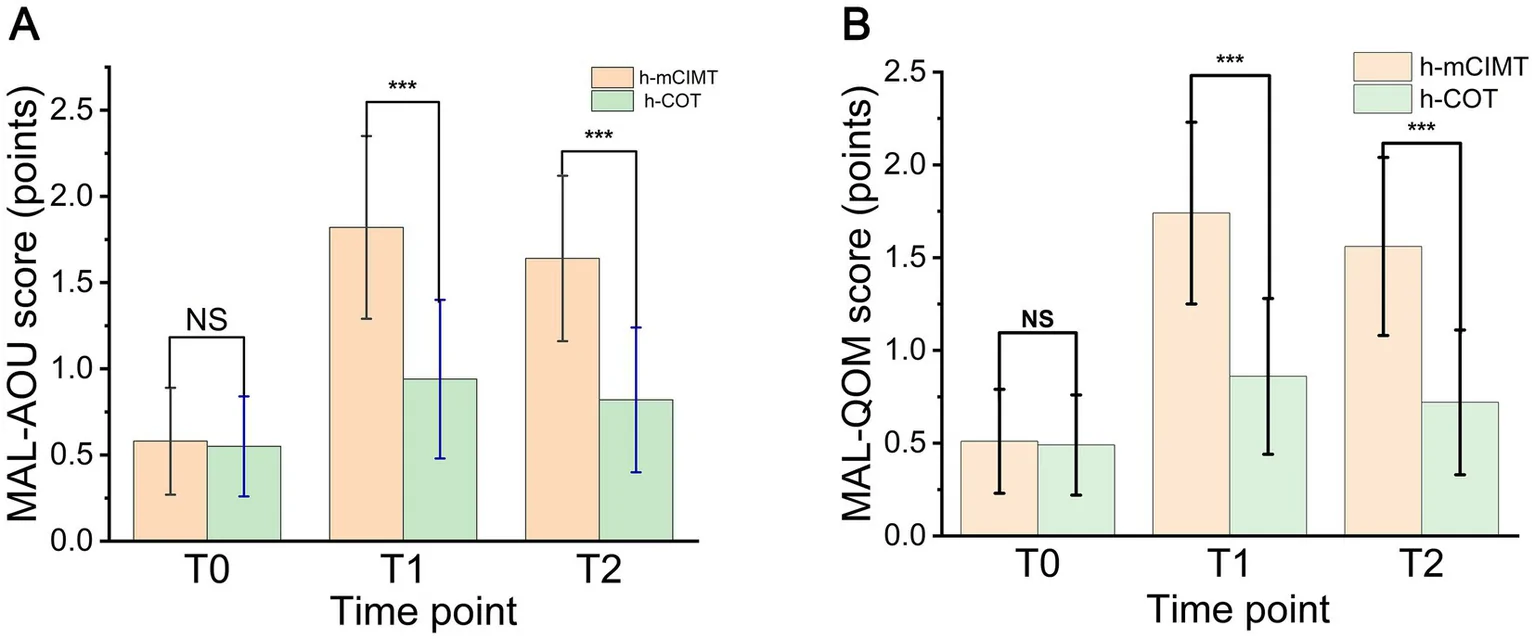
Temporal changes in MAL-AOU and MAL-QOM scores in the two cohorts.

Real-world use of the affected upper limb, assessed by the MAL, improved more substantially in the h-mCIMT cohort than in the h-COT cohort (Table 5, Figure 2). At T1, both the amount of use (AOU) and quality of movement (QOM) subscales showed significantly greater gains in the h-mCIMT cohort, with identical between-cohort differences of 0.88 points for AOU and QOM (both *p* < 0.001). These differences were not only statistically significant but also functionally relevant, indicating that h-mCIMT was associated with more frequent and better-quality use of the affected hand in daily life. Importantly, this advantage persisted at the 3-month follow-up. Although MAL scores declined slightly after the end of the structured rehabilitation period, AOU and QOM scores in the h-mCIMT cohort remained clearly higher than baseline and significantly higher than those in the h-COT cohort at T2 (both *p* < 0.001). In contrast, the h-COT cohort showed more limited improvement and a greater tendency toward decline after the rehabilitation period. GEE analysis confirmed significant cohort-by-time interactions for both MAL-AOU and MAL-QOM (Wald *χ*^2^ = 46.258 and 48.624, respectively; both *p* < 0.001), supporting a sustained association between h-mCIMT following BoNT-A injection and spontaneous use of the affected upper limb in everyday activities.

**Table 5:** Comparison of MAL scores between the two cohorts at three time points.

MAL scores	h-mCIMT cohort (*n* = 49)	h-COT cohort (*n* = 49)	Between-cohort *p* value
MAL-AOU (score)			
T0	0.58 ± 0.31	0.55 ± 0.29	0.620
T1	1.82 ± 0.53	0.94 ± 0.46	<0.001
T2	1.64 ± 0.48	0.82 ± 0.42	<0.001
MAL-QOM (score)			
T0	0.51 ± 0.28	0.49 ± 0.27	0.717
T1	1.74 ± 0.49	0.86 ± 0.42	<0.001
T2	1.56 ± 0.48	0.72 ± 0.39	<0.001

### 3.6 Safety evaluation

No serious adverse events related to BoNT-A injection or home-based rehabilitation occurred during the study period (Table 6). Safety analysis included all 102 patients initially classified into the two rehabilitation cohorts, regardless of follow-up completeness. The overall incidence of adverse events was low and comparable between the h-mCIMT and h-COT cohorts (11.8% vs. 9.8%; *p* = 0.751). All reported events were mild and transient and resolved spontaneously or after simple adjustment of training intensity. The observed safety profile was consistent with the expected effects of BoNT-A injection and home-based upper-limb rehabilitation.

**Table 6:** Comparison of adverse events between the two cohorts.

Adverse event	h-mCIMT cohort (*n* = 51)	h-COT cohort (*n* = 51)	*p* value
Injection-related adverse events			
Transient injection-site pain	3 (5.9)	2 (3.9)	1
Brief weakness in finger extension, probably injection-related	1 (2.0)	0 (0.0)	1
Training-related adverse events			
Mild fatigue of the affected upper limb during the early stage of training	3 (5.9)	0 (0.0)	0.243
Mild pain induced by stretching exercises	0 (0.0)	2 (3.9)	0.495
Soreness and distending discomfort of upper-limb muscles	0 (0.0)	1 (2.0)	1
Total number of adverse events	7	5	–
Total number of participants with adverse events [*n* (%)]	6 (11.8)	5 (9.8)	0.751
Serious adverse events	0 (0.0)	0 (0.0)	–

The single case of brief weakness in finger extension was recorded as an injection-related event and was considered a probable local pharmacological effect of BoNT-A rather than behavioral fatigue from training, although formal causality assessment was not performed. Injection-site pain was self-limiting, and early training-related fatigue improved after brief modification of session intensity; the finger-extension weakness resolved without additional intervention. These findings suggest that h-mCIMT initiated after BoNT-A injection was feasible and well tolerated in routine clinical practice, without evidence of increased adverse-event risk compared with conventional home-based occupational therapy.

### 3.7 Potential mechanism speculation

**Figure 3:**
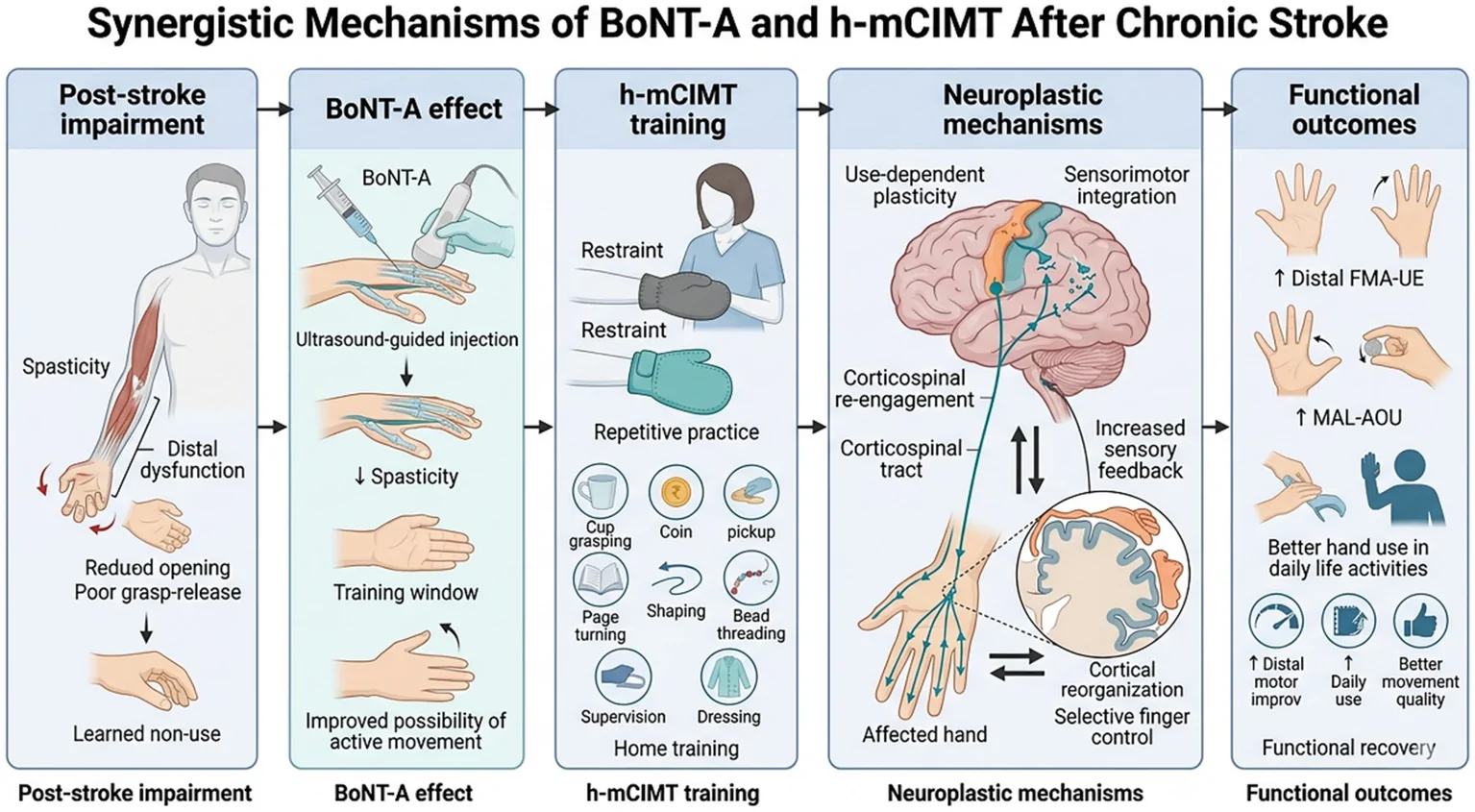
Potential mechanism speculation underlying the superior effects of BoNT-A plus home-based modified constraint-induced movement therapy after chronic stroke.

To illustrate the potential mechanisms underlying the additional functional gains observed in the h-mCIMT cohort after BoNT-A injection, a schematic summary is presented in Figure 3. The selective improvement in distal FMA-UE scores and real-world arm use suggests that the observed benefit of h-mCIMT was not driven solely by spasticity reduction. In both cohorts, MAS scores decreased after BoNT-A injection, with no significant between-cohort differences, indicating comparable pharmacological effects. The additional improvements observed in the h-mCIMT cohort may therefore reflect the interaction between pharmacological tone reduction and use-dependent motor relearning. BoNT-A may have created a temporary therapeutic window by reducing excessive finger flexor activity, thereby allowing more efficient voluntary hand opening, grasp release, and task practice. Within this window, h-mCIMT encouraged repeated use of the affected hand while limiting compensatory reliance on the unaffected limb. This approach directly targets learned non-use, increases task-specific sensorimotor input, and reinforces goal-directed distal movements. The shaping strategy, in which task difficulty was continuously adjusted according to patient performance, may further facilitate corticospinal engagement and progressive refinement of selective finger control. The sustained advantage in MAL scores indicates that these motor gains extended beyond structured rehabilitation into daily behavior. Taken together, h-mCIMT may function as a behavioral amplifier of BoNT-A treatment, facilitating the translation of reduced muscle tone into meaningful functional recovery through repeated, context-relevant practice.

## 4 Discussion

### 4.1 Principal findings

In patients with chronic stroke and finger flexor spasticity, those receiving h-mCIMT shortly after BoNT-A injection showed greater recovery of distal upper-limb motor function than those receiving dose-matched h-COT. The main difference was observed in the distal FMA-UE subscore, which was the primary outcome of this study. The h-mCIMT cohort showed a larger improvement after the initial 4-week rehabilitation phase, and this advantage was maintained at the 3-month follow-up. In contrast, proximal FMA-UE subscores did not differ significantly between cohorts, indicating that the observed functional benefit was not generalized across the entire upper limb but was concentrated in the wrist and hand, which were the principal functional targets of the intervention (18). This finding is clinically relevant because distal hand recovery in chronic stroke remains particularly difficult, especially when finger flexor spasticity interferes with hand opening, grasp release, and object manipulation (19, 20). Although BoNT-A is widely used to reduce focal spasticity, functional improvement after injection remains inconsistent. The present findings suggest that the functional value of BoNT-A may depend on active, task-oriented motor relearning during the post-injection therapeutic window (5).

### 4.2 Relationship between spasticity reduction and motor recovery

Both cohorts obtained comparable relief of finger flexor spasticity after BoNT-A injection, but only the h-mCIMT cohort demonstrated greater gains in distal motor function and real-world arm use (21). These findings suggest that h-mCIMT may not simply enhance the antispastic effect of BoNT-A; rather, it may facilitate the translation of reduced muscle tone into functional recovery through repeated, task-oriented use of the affected hand. This interpretation is supported by the structure of the rehabilitation programme. In routine clinical practice, rehabilitation was typically initiated around day 3 after injection and was performed for approximately 60 min per day, 5 days per week, over 4 weeks. Patients practiced progressive gross motor tasks, fine motor tasks, and activities of daily living while the unaffected hand was temporarily restrained. The shaping strategy, in which task difficulty was continuously adjusted according to performance, may have encouraged repeated use of the paretic hand at an appropriate challenge level. In this context, BoNT-A may have created a temporary biomechanical window by reducing excessive finger flexor activity, while h-mCIMT may have provided the behavioral support needed to facilitate selective distal control. The sustained improvement in MAL-AOU and MAL-QOM further supports this interpretation. However, the discrepancy between the significant MAL findings and the non-significant ARAT result requires cautious interpretation. Because participants and caregivers were not blinded, expectancy effects, reporting bias, and a possible Hawthorne effect may have influenced the interview-based MAL scores. Therefore, these findings indicate greater patient-reported use of the affected limb rather than objectively confirmed improvement in upper-limb activity.

### 4.3 Importance and innovation of this study

An important strength of this study lies in the integration of BoNT-A injection with a structured, home-based rehabilitation strategy. Previous clinical practice has often separated spasticity management from functional rehabilitation. In contrast, the present study linked the pharmacological target and the rehabilitation target: ultrasound-guided BoNT-A was delivered to the finger flexor muscles, and h-mCIMT subsequently focused on repeated use of the affected hand through tasks directly related to distal motor function and daily activity. Another important feature is the home-based rehabilitation model (22). Intensive upper-limb rehabilitation is often difficult to sustain in routine outpatient settings, especially for patients in the chronic phase. This study incorporated caregiver training, daily logs, weekly WeChat video follow-up, and uploaded training videos to improve treatment fidelity outside the hospital. The low dropout rate and absence of serious treatment-related adverse events suggest that this approach was feasible and safe. Thus, the present findings provide not only evidence supporting a combined therapeutic strategy, but also a practical model for delivering post-BoNT-A rehabilitation in real-world clinical settings.

### 4.4 Limitations and future directions

Several limitations should be acknowledged. First, this was a single-center retrospective cohort study, and the findings should be confirmed in larger multicentre prospective studies. Second, although baseline characteristics were comparable between cohorts, residual confounding and selection bias inherent to retrospective analyses cannot be completely excluded. Treatment assignment was based on routine clinical decision-making rather than randomized allocation, which may have influenced rehabilitation exposure. Third, the inclusion criteria required preserved cognition, residual upper-limb function, independent sitting ability, and caregiver support; therefore, the findings may not be generalizable to patients with more severe impairment or limited family resources. Because the distal FMA-UE includes voluntary wrist-control items, unmeasured residual wrist flexor spasticity may have influenced the primary outcome and limits attribution of the observed improvement specifically to finger motor recovery. In addition, the follow-up period was limited to 3 months, and the long-term durability of the observed functional improvements remains uncertain. Future studies should examine whether repeated BoNT-A plus h-mCIMT cycles are associated with cumulative benefits and whether digital monitoring can further improve adherence (23). Neurophysiological, kinematic, and real-world activity measures should also be incorporated to clarify mechanisms and identify patients most likely to benefit.

## 5 Conclusion

In this retrospective cohort study, BoNT-A combined with home-based modified constraint-induced movement therapy was associated with improved distal upper-limb motor function in chronic stroke. This combined strategy was also associated with greater real-world use of the affected hand. The h-mCIMT cohort showed additional functional gains despite similar reductions in spasticity between groups. This suggests that structured, task-oriented use of the paretic hand may help translate spasticity improvement into functional recovery. Importantly, the rehabilitation strategy was feasible in a home setting, supported by caregiver training, remote follow-up, and video-based quality control, and was not associated with increased safety concerns. These findings support a shift from viewing post-stroke spasticity as an isolated impairment toward a more integrated, function-oriented rehabilitation strategy. Larger prospective multicentre studies with longer follow-up and mechanistic assessments are warranted to further validate these observations.

## Data Availability

The original contributions presented in the study are included in the article/supplementary material, further inquiries can be directed to the corresponding author.
